# A List of Medical Offers of Service

**Published:** 1915-07-10

**Authors:** Thomas H. Bickerton

**Affiliations:** Liverpool.


### THE EDITOR'S LETTER-BOX.

### A List of Medical Offers of Service.

Sir,—In your issue for June 5, 1915, there is an article entitled "The Shortage in Hospital Medical Staffs," and on p. 206 is a paragraph which reads as follows: ". . . A considerable number of medical man of all ages, qualifications, and experience have sent in their names to the War Office offering service in the existing emergency. . . . A list, we understand, of these offers has been made at the War Office. . . ."

As the Secretary of the Medical Board of the Liverpool Royal Infirmary, I am writing to ask if you can tell me to whom application should be made in order to obtain a copy of the above-mentioned list?—I am, yours faithfully,

Liverpool. Thomas H. Bickerton.

[Applications should be addressed to Sir Reginald Brade, K.C.B., and to Surgeon-General Keogh, K.C.B., the War Office, London.—Ed. The Hospital.]

